# One-year changes in axial length and refraction in children using low-level red light and distant-image screen for myopia control: a randomized controlled trial

**DOI:** 10.3389/fmed.2025.1542620

**Published:** 2025-03-25

**Authors:** Ke Yang, Yuhan Wang, Xiaoxia Li, Sumeng Liu, Hui Shi, Liya Qiao

**Affiliations:** ^1^Beijing Tongren Eye Center, Beijing Key Laboratory of Ophthalmology and Visual Science, Department of Ophthalmology, Beijing Tongren Hospital, Capital Medical University, Beijing, China; ^2^Department of Ophthalmology, Beijing Shijitan Hospital, Capital Medical University, Beijing, China

**Keywords:** myopia control, red light therapy, distant-image therapy, axial length reduction, myopia regression

## Abstract

**Objective:**

To assess the efficacy and safety of 650-nm low-level red light (RL) and distant-image therapy (DIT) for myopia.

**Methods:**

A randomized clinical trial. Children aged 8–10 years with a spherical equivalent error (SER) ranging from −1 to −1.5 diopters (D) were enrolled, and were randomly allocated to the following group: RL, DIT, RL + DIT, and control in a 1:1:1:1 ratio. The primary outcomes were changes in SER and axial length (AL).

**Results:**

One hundred and sixteen children randomized, girls accounted for 45.69% (53/116). The median one-year changes in SER were 0.21D (inter-quartile range, IQR: −0.03D to 0.46D), −0.06D (−0.32D to 0.19D), −0.08D (−0.31D to 0.14D), and −0.30D (−0.51D to −0.09D), respectively, for the RL + DIT, RL, DIT, and the control group. The median one-year changes in AL were 0.04 mm (−0.03 mm to 0.13 mm), 0.05 mm (−0.03 mm to 0.14 mm), 0.30 mm (0.22 mm to 0.37 mm), and 0.42 mm (0.35 mm to 0.49 mm), respectively, for the RL + DIT, RL, DIT, and the control group. Fundus photographs revealed no retinal changes across all groups.

**Conclusion:**

Participants who underwent daily 650-nm low-level red light therapy combined with distant-image screen intervention for 12 months demonstrated a significant deceleration in myopia progression, with 79.3% exhibiting potential for reversal of myopia. No safety concerns were identified through OCT and fundus photography.

**Clinical trial registration:**

ClinicalTrials.gov, NCT06683287.

## Introduction

Myopia has become a major public health issue worldwide, especially in China and other Asian countries, where the prevalence of myopia among young people is steadily increasing. The advancement of myopia is linked to a variety of factors, among which extended close-up work in poor lighting conditions is regarded as a primary contributor (1). This pattern of visual activity results in accommodative lag, thinning of the choroid and sclera, and ultimately leads to axial length elongation and myopia progression. Meta-analyses have suggested that the progression of myopia in children is closely correlated with the amount of near work they engage in at home post-school hours. This correlation exists because the home environment often involves prolonged periods of close-up work, and Asian children, burdened with significant academic demands post-school, tend to spend extended periods engaged in such activities, contributing to an early and high incidence of myopia (2).

For a long time, many researchers have been trying to find ways to control myopia. At present, there are some effective measures in clinic, such as atropine and orthokeratology. In recent years, there have been two new intervention methods, low-level red light (RL) and distant-image screen (DIT). Multiple studies have reported that RL has a remarkable effect on myopia control (3–5), however, DIT remains a novel concept for both researchers and patients to some extent. Theoretically, through optical design, DIT projects the image of an object on the screen to a distance of 5 meters or more. In this case, although the child sits in front of the screen, he or she does not use the eye at close range (6). It remains unclear whether myopia progresses slower if children use DIT.

This study aims to compare the efficacy of RL, DIT, and their combination for myopia control.

## Methods

This is a single-center, randomized controlled clinical trial. Children with myopia ranging from −1 to −1.5 D were enrolled. They were assigned to the control, or they received interventions of RL, DIT, or a combination of both.

The efficacy of different interventions was evaluated by comparing changes in primary outcomes, including axial length (AL) and spherical equivalent error (SER).

### Setting

Participants were recruited from the Huilongguan School, Yu Xiang Primary School in Beijing, China. All recruited children were invited to the outpatient department of Beijing Tongren Hospital for eligibility assessment. Participants were asked to finish a series ophthalmic examinations. Participants were randomly assigned in a 1:1:1:1 ratio to the RL + DIT group, RL group, DIT group, or the control group. The recruitment period was from April 10, 2023, to May 15, 2023.

### Human ethics and consent to participate declarations

The study has been approved by the Ethics Committee of Beijing Tongren Hospital, Capital Medical University. This clinical trial adheres to the principles of the Declaration of Helsinki. The study follows the Consolidated Standards of Reporting Trials (CONSORT) reporting guidelines. The present trial was registered in ClinicalTrials.gov. Number: NCT06683287. Register date: November 08, 2024.

### Inclusion and exclusion criteria

#### Inclusion criteria

1. Age between 8 and 10 years old.
2. The cycloplegic spherical equivalent error after pupil dilation was between −1.0 D and −1.5D.
3. Absolute value of corneal astigmatism ≤1.25D.
4. Absolute value of interocular refraction discrepancy ≤1.5D.
5. Near-distance exophoria <10 prism diopters (Δ) and far-distance exophoria <6 prism diopters (Δ).
6. Willingness to participate in the study and signed informed consent (children and parents both need to sign).

#### Exclusion criteria

1. Intraocular pressure was below 10 mmHg or higher than 22 mm Hg.
2. Presence of amblyopia, or ocular pathological conditions such as retinal, lens, or corneal disorders.
3. Children currently using other interventions for myopia control, including but not limited to atropine or orthokeratology.
4. Patients with systemic and immune disorders such as albinism, psoriasis, nephrotic syndrome, systemic lupus erythematosus, diabetes, etc.
5. Individuals with conditions like Tourette’s syndrome or epilepsy.

### Intervention and study procedures

Participants in the RL + DIT and RL groups received a RL apparatus (LS-03B; Yishiliang Inc.), with a power output of 0.39 mW, which was certified as a Class II medical device by the provincial medical supervision departments of China. They were directed to utilize this apparatus twice a day for 3-min sessions, ensuring a gap of over 4 h between each use. A DIT (RIO-Max2.0; Ruishi Inc.) would be given to children in either the RL + DIT or DIT group, children were asked to use this device for ≥1 h/day. Distant image screen uses virtual telephoto technology to project the image on the screen to 5 meters away, so although the placement of the screen is the same as that of general electronic products like computer, the image seen is 5 meters away (there are examples of technical principles in the Supplementary Figure 1). Across all groups, children wear single-vision spectacle lenses. For control group, no other intervention was given. The RL and DIT device would be automatically connected to the internet when powered on, facilitating precise tracking of usage duration. Intervention adherence is determined by dividing the actual usage duration by the prescribed usage duration.

### Outcomes and measurements

Primary outcomes include changes in AL and cycloplegia SER. SER was calculated from the dioptric powers of the sphere and half of the cylinder. The children’s pupils were dilated using tropicamide eyedrops (Producer: Shenyang Xingqi Eye Medicine Co., Ltd. Usage: One drop of 0.05 mL each time, a total of three times before optometry, with 10 min interval between each drop.), then SER was subsequently measured using an auto refractor (ARK-510A, Nidek Co., Ltd.). AL was measured using an optical biometer (Colombo IOL, Moptim). The two primary outcomes (changes in AL and changes in cycloplegia SER) and secondary outcomes were determined at 12-month follow-up visit.

For safety evaluation, intra-ocular pressure, fundus photography and optical coherence tomography (OCT) were performed. We especially paid attention to hemorrhage, exudation, retinal nerve fiber layer defect, and discontinuity of retina layer. Fundus photography was performed with a Canon retinal fundus camera (CR-DGI; Canon Inc.). The interpretation of the fundus images was performed independently by two ophthalmologists from Beijing Tongren Hospital, and the final interpretation was given by another senior ophthalmologist (LQ). The intra-ocular pressure was measured by a noncontact tonometer (Canon TX-20; Canon Inc.).

### Sample size estimation

The sample size calculation method is as follows:


$$N_{ij}=\frac{{\left(Z_{1-\alpha /2T}+Z_{1-\beta }\right)}^{2}\;\left(\sigma_{i}^{2}+\sigma_{j}^{2}\right)}{\delta_{ij}^{2}}$$

$$N=\max\left(N_{ij}\right)$$

*N_ij_* represents the sample size of each group calculated from data based on the *i*th and *j*th groups. $Z_{1-\alpha /2}$ is 1.96 at 0.05 significance level (2.36 for 3 times comparison) and $Z_{1-\beta }$ is 0.84 at a power of 0.8. *σ_i_* represents the standard deviation of primary outcome in the first group. *σ_j_* represents the standard deviation of primary outcome in the second group. δ*_ij_* represent the difference in primary outcome between two groups. *T* represents the number of comparisons.

According to the preliminary experimental results, the minimum difference between the three experimental groups and the control group was 0.22 ± 0.23D. Besides, we consider a within 20% loss-to follow-up rate, thus the sample size was determined to be 29 for each group.

### Statistical analysis

For continuous variables, data are expressed as mean ± standard deviation (SD) if they are normally distributed. Otherwise, data are expressed as median and inter-quartile range (IQR). For categorical data, counts with percentages are presented. Analysis of variance (ANOVA) is used to compare the statistical differences in continuous data between four treatment groups. The chi-square test is performed for the comparison of the categorical results. All post-hoc comparisons between treatment groups are adjusted with Bonferroni correction. Per the study protocol, participants were restricted to single-vision spectacle lenses for daily activities, with explicit prohibition of additional vision correction modalities beyond the prescribed interventions. However, it was observed that some children used orthokeratology lenses across all groups. This protocol deviation may introduce confounding variables requiring statistical adjustment to mitigate potential biases in intervention efficacy assessment. To adjust the confounding effect, the baseline measurement and the use of ortho-k were included in the mixed model as covariates. Least square means were calculated using the mixed model. *p* < 0.05 is considered as statistically significant.

## Results

### Participant screening

**Figure 1:**
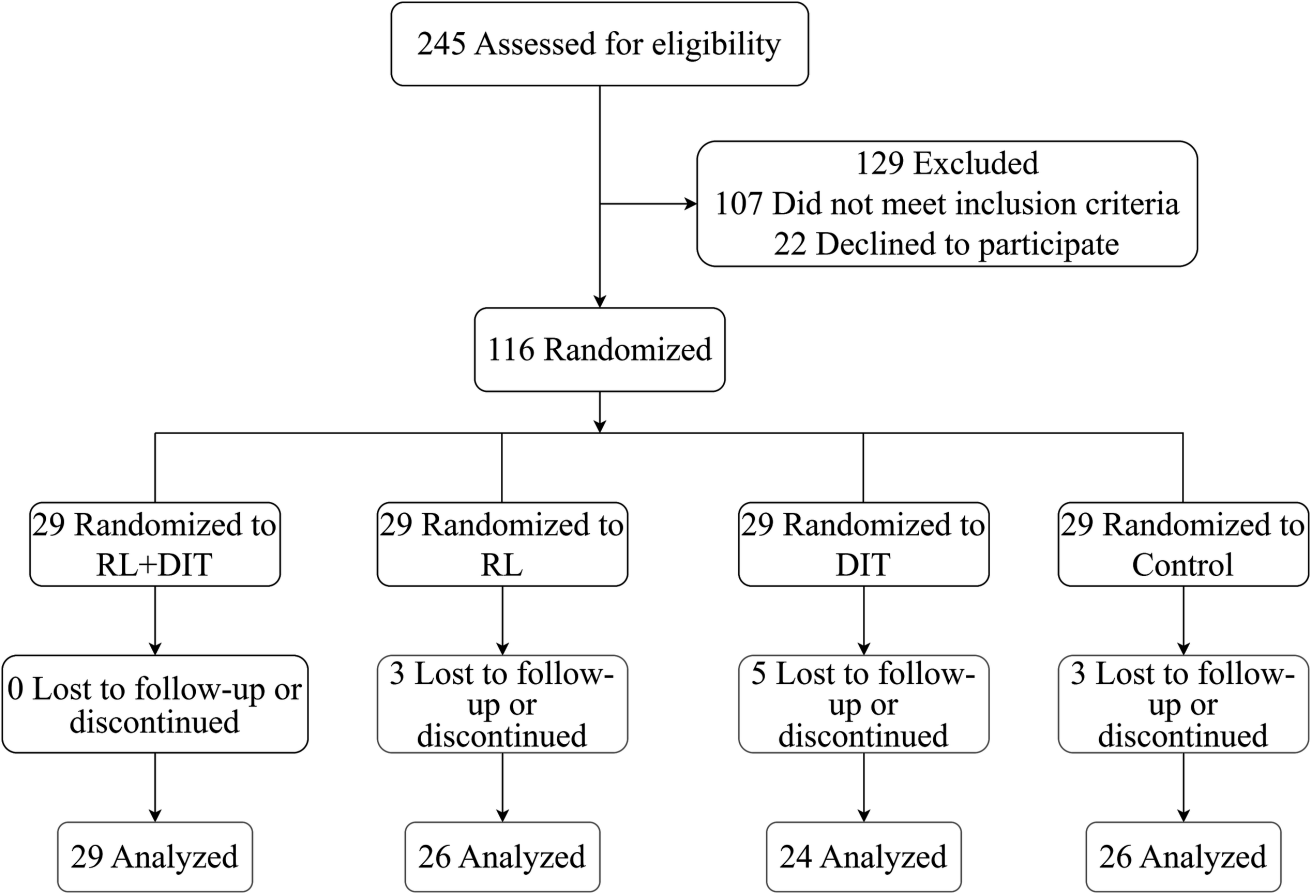
Flow chart of participants selection.

Initially, 245 registered children were assessed for eligibility, 107 children did not meet the inclusion criteria, and 22 refused to sign the informed consent, 116 children participated in the study eventually. The loss to follow-up rates were 0, 10.3% (3/ 29), 17.2% (5/29) and 10.3% (3/29) for the RL + DIT group, RL group, DIT group and control group at 12 months (Figure 1).

### Participants’ baseline characteristics

The median (IQR) age was 9.0 years (8 years to 10 years), 9.0 years (8 years to 10 years), 9.0 years (8 years to 10 years), and 9.0 years (8 years to 10 years) for the RL + DIT group, the RL group, the DIT group, and the control group. There were 17 (58.6%) girls in the RL + DIT group, 10 girls (38.5%) in the RL group, 13 (54.2%) girls in the DIT group, 13 (50%) in the control group. The median SER was −1.25D (IQR: −1.38D to −1.13D), −1.13D (−1.38D to −1.00D), −1.13D (−1.25D to −1.00D), and −1.13D (−1.25D to −1.00D) for the RL + DIT group, the RL group, the DIT group, and the control group. The mean AL was 24.07 mm ± 0.71 mm, 24.30 mm ± 0.87 mm, 23.99 mm ± 0.85 mm, and 23.93 mm ± 0.66 mm for the RL + DIT group, the RL group, the DIT group, and the control group. The details of the participants’ baseline information are shown in Table 1.

**Table 1:** Children’s demographic characteristics and ocular parameters at baseline.

Variables	RL + DIT	RL	DIT	Control	*p*
Gender					0.493
Girls	17 (58.6%)	10 (38.5%)	13 (54.2%)	13 (50.0%)	
Boys	12 (41.4%)	16 (61.5%)	11 (45.8%)	13 (50.0%)	
Total	29	26	24	26	
Anterior chamber depth (mm)	3.89 ± 0.74	3.91 ± 0.34	3.72 ± 0.28	3.75 ± 0.20	0.322
Axial length (mm)	24.07 ± 0.71	24.30 ± 0.87	23.99 ± 0.85	23.93 ± 0.66	0.351
Central corneal thickness (um)	537.59 ± 27.84	544.54 ± 27.96	540.80 ± 31.01	543.08 ± 22.47	0.799
K1 (D)	42.53 ± 1.41	42.38 ± 1.47	42.30 ± 1.39	42.68 ± 1.30	0.781
K2 (D)	43.56 ± 1.68	43.30 ± 1.53	43.36 ± 1.43	43.59 ± 1.38	0.873
Length thickness (mm)	3.36 (3.24–3.55)	3.28 (3.08–3.47)	3.35 (3.28–3.41)	3.44 (3.22–3.58)	0.243
Spherical equivalent error (D)	−1.25 (−1.38 to −1.13)	−1.13 (−1.38 to −1.00)	−1.13 (−1.25 to −1.00)	−1.13 (−1.25 to −1.00)	0.220
Visual acuity (LogMAR)	0.10 (0.10–0.30)	0.10 (0.10–0.20)	0.10 (−0.10 to 0.20)	0.10 (−0.10 to 0.20)	0.337
Age (years)	10.00 (8.00–10.00)	9.00 (8.00–10.00)	9.00 (8.00–10.00)	9.00 (8.00–10.00)	0.253

RL, red light; DIT, distant-image therapy; DIT + RL, red light + distant-image therapy. LS means is calculated using mixed model adjusting baseline value and use of orthokeratology.

### Intervention compliance

The RL and DIT device was automatically connected to the internet once powered on, and thus, the duration of use could be accurately recorded. Without counting the 11 children who quit the trial or were lost to follow-up, the compliance rate for the RL use and DIT use exceeds 80% (Both the RL and DIT devices are equipped with a backend recording function, capable of logging the usage duration of the subjects. By performing a division operation using the recorded usage duration and the expected usage duration, the compliance rate can be computed. This aspect has been explicitly stated in our revised manuscript).

### Changes in AL and refractive status

**Figure 2:**
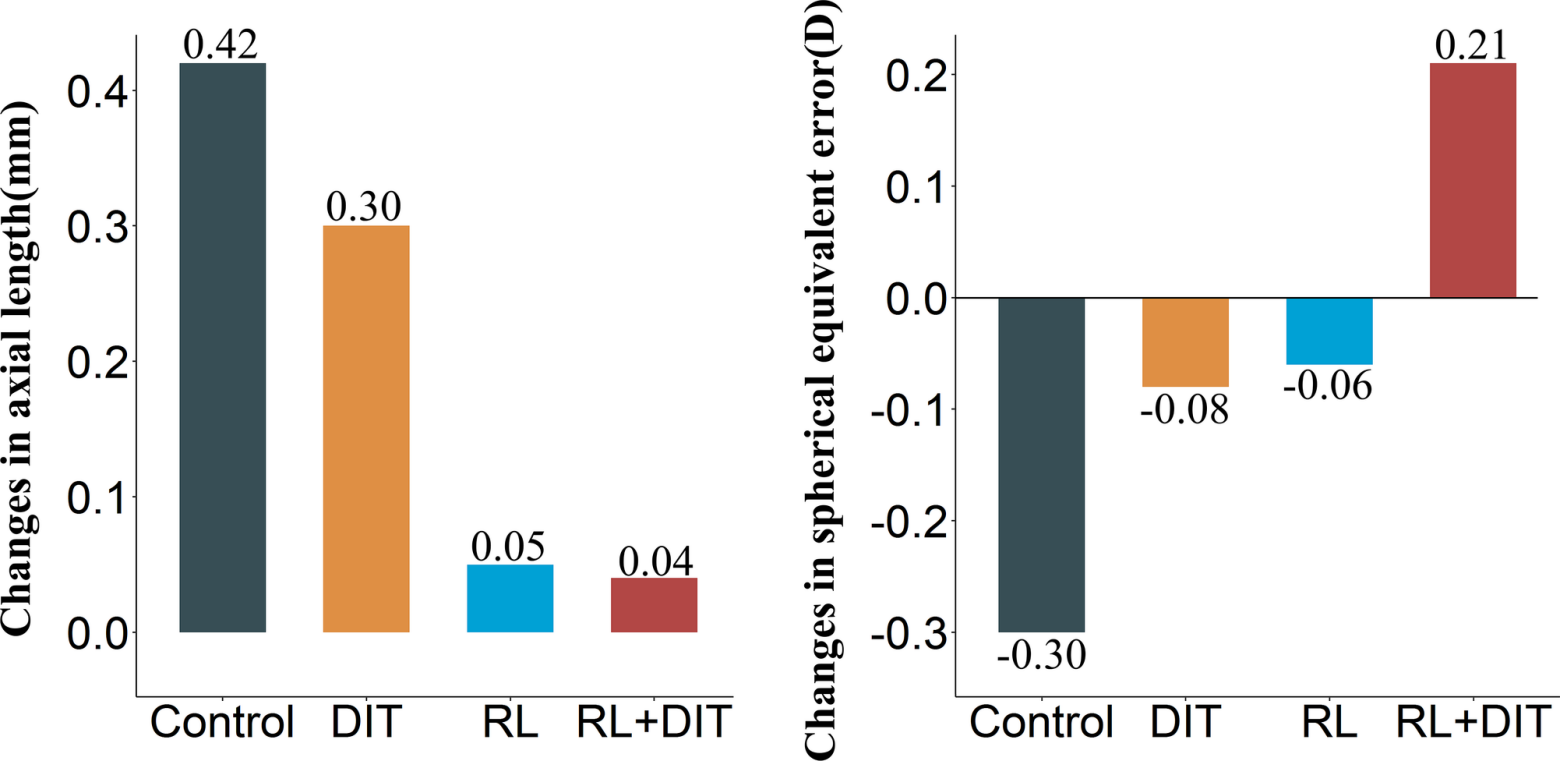
One-year changes in primary outcomes.

The one-year median (IQR) changes in SER were 0.21D (−0.03D to 0.46D), −0.06D (−0.32D to 0.19D), −0.08D (−0.31D to 0.14D), −0.30D (−0.51D to −0.09D) for the RL + DIT group, RL group, DIT group, and control group, respectively (Table 2). Compared with the control, there was significant difference with the RL + DIT group (*t* = 7.216, *p* < 0.001), RL group (*t* = 4.331, *p* < 0.001), DIT group (*t* = 4.279, *p* < 0.001), and control group (Figure 2).

**Table 2:** One-year changes in spherical equivalent error and axial length by treatment group.

Outcomes	Intervention	LS means (95% CI)	*p*	*p*1 (compared with control)
Spherical equivalent error (D)	RL + DIT	0.21 (−0.03 to 0.46)	0.085	<0.001
	RL	−0.06 (−0.32 to 0.19)	0.615	<0.001
	DIT	−0.08 (−0.31 to 0.14)	0.461	<0.001
	Control	−0.30 (−0.51 to −0.09)	0.005	—
Axial length (mm)	RL + DIT	0.04 (−0.03 to 0.13)	0.235	<0.001
	RL	0.05 (−0.03 to 0.14)	0.218	<0.001
	DIT	0.30 (0.22 to 0.37)	<0.001	<0.001
	Control	0.42 (0.35 to 0.49)	<0.001	—

RL, red light; DIT, distant-image therapy; DIT + RL, red light + distant-image therapy. LS means is calculated using mixed model adjusting baseline value and use of orthokeratology.

The one-year median (IQR) changes in AL were 0.04 mm (−0.03 mm to 0.13 mm), 0.05 mm (−0.03 mm to 0.14 mm), 0.30 mm (0.22 mm to 0.37 mm), and 0.42 mm (0.35 mm to 0.49 mm) for the RL + DIT group, RL group, DIT group, and control group, respectively (Table 2). Compared with the control, there was significant difference with the RL + DIT group (*t* = −7.776, *p* < 0.001), RL group (*t* = −7.138, *p* < 0.001), DIT group (*t* = −3.562, *p* < 0.001), and control group (Figure 2).

### Other outcomes

At 12 months, the RL + DIT group had the highest proportion of children showing a shortened axial length (AL) of 0.02 mm/month or more (50.0%), while the control group showed no such cases. Conversely, the control group had the highest proportion of children with an elongated AL of ≥0.02 mm/month (100.0%), followed by the DIT group (87.5%). In terms of myopization, the control group had the highest percentage of children showing myopic changes (61.5%), whereas the RL + DIT group had the lowest (10.3%) (Table 3). In the RL + DIT group, one case of −1.0D myopia reverted to emmetropia.

**Table 3:** Comparison of changes in spherical equivalent error and axial length by treatment group.

Outcomes	Group	RL + DIT	RL	DIT	Control	*p*
Axial length (mm)/monthly (12 months)	Elongated ≤0.02 mm/month	2 (7.7%)	8 (27.6%)	1 (4.2%)		<0.001
	Elongated >0.02 mm/month	8 (30.8%)	11 (37.9%)	21 (87.5%)	26 (100.0%)	
	Shortened	13 (50.0%)	7 (24.1%)	2 (8.3%)		
	Unchanged	3 (11.5%)	3 (10.3%)			
Axial length (mm)/monthly (6 months)	Elongated ≤0.02 mm/month	7 (24.1%)	7 (26.9%)	10 (41.7%)	9 (34.6%)	<0.001
	Elongated >0.02 mm/month			10 (41.7%)	17 (65.4%)	
	Shortened	17 (58.6%)	15 (57.7%)	2 (8.3%)		
	Unchanged	5 (17.2%)	4 (15.4%)	2 (8.3%)		
Myopic cure (12 months)	No	28 (96.6%)	26 (100.0%)	24 (100.0%)	26 (100.0%)	1.000
	Yes	1 (3.4%)				
Spherical equivalent error (D) (12 months)	Hyperopic shift	23 (79.3%)	18 (69.2%)	12 (50.0%)	7 (26.9%)	<0.001
	Myopic shift	3 (10.3%)	8 (30.8%)	8 (33.3%)	16 (61.5%)	
	Unchanged	3 (10.3%)		4 (16.7%)	3 (11.5%)	
Spherical equivalent error (D) (6 months)	Hyperopic shift	25 (86.2%)	16 (61.5%)	14 (58.3%)	13 (50.0%)	0.023
	Myopic shift	2 (6.9%)	6 (23.1%)	9 (37.5%)	11 (42.3%)	
	Unchanged	2 (6.9%)	4 (15.4%)	1 (4.2%)	2 (7.7%)	

RL, red light; DIT, distant-image therapy; DIT + RL, red light + distant-image therapy. Shortened axial length is defined as the axial length at 6 months or 12 months is 0.02 mm shorter than baseline. Elongated axial length is defined as the axial length at 6 months or 12 months is 0.02 mm longer than baseline. Unchanged axial length is defined as the change of axial length at 6 months or 12 months is no more than 0.02 mm compared with baseline. Axial length elongated per month = (Axial length at 6 months or 12 months − Axial length at baseline)/Axial length at 6 months or 12 months.

The average changes in choroid thickness measured at the fovea of the macula were 15 μm ± 14 μm, 12 μm ± 12 μm, −17 μm ± 19 μm, and −31 μm ± 26 μm, for the RL + DIT group, RL group, DIT group, and control group. Significant difference was found between the RL + DIT group and the control (*t* = 10.256, *p* < 0.001), between the RL group and the control (*t* = 9.778, *p* < 0.001), between the DIT group and the control (*t* = 5.621, *p* < 0.001).

Eye fundus images indicated no hemorrhage, exudation, or retinal nerve fiber layer defect for any child. No discontinuity of retina layer was found by OCT images.

## Discussion

The present study revealed that the observed differential efficacy between intervention modalities raises critical questions regarding the synergistic mechanisms of combined photobiomodulation and optical defocus strategies. Notably, the RL + DIT cohort exhibited a median SER progression of +0.21 D compared to −0.06 D (RL alone) and −0.08 D (DIT alone), suggesting potential additive effects that warrant mechanistic investigation. The safety profile, though reassuring, necessitates cautious interpretation. While no acute retinal damage was detected through standard imaging protocols, the long-term biological consequences of chronic RL exposure on mitochondrial DNA integrity remain uncharacterized.

There are several effective methods for myopia control before RL and DIT, such as orthokeratology (7, 8), atropine eye drops (9, 10), peripheral defocus-modifying lenses (11, 12) and outdoor time (13). Atropine was reported to be one of the most effective therapy options (14, 15). However, atropine treated children were still reported to experience a progress in SER of up to −0.63D per year. In contrast, the one-year change in the SER in children receiving daily 650 nm RL and DIT intervention in the present study was 0.21D per year, and were −0.06D for single RL use, and were −0.08 D for single DIT use. Based on previously mentioned evidence, the effect of 650 nm RL, and DIT, as well as their combination, may be more potent than that of other available interventions (16). However, the present trial did not directly compare various intervention measures, and further evidence is needed to support this conclusion.

Prolonged near work can lead to accommodative lag, which is associated with thinning of the choroid and sclera, and elongation of the AL (17). These changes are considered to be the primary stimuli for the progression of myopia (17, 18). The DIT effectively shifts the visual environment from a near-vision focus to a far-vision perspective, alleviating the accommodative lag and reducing ocular strain, which are instrumental in curbing the advancement of myopia (6, 19). By incorporating DIT into the regular visual activities of children, including study and leisure, there is an opportunity for extended exposure to distant visual stimuli. This extended exposure could potentially result in a hyperopic shift, fostering an increase in choroidal thickness and a reduction in AL, which are beneficial in the context of myopia management (20). However, in the present study, the two groups receiving RL intervention demonstrated significant choroidal thickening, whereas the DIT group exhibited observable thinning, the degree of thinning was significantly lower than that of the control group. These differences align with the differential AL progression observed across groups. These findings suggest that DIT may be suboptimal for established myopia management, at least not recommended for myopia control alone, but maybe a feasible prevention strategy for pre-myopic children.

Employing DIT over an extended duration may help reduce in the curvature of the crystalline lens, alleviating the accommodational stress within the eye. This alleviation is likely to be a significant factor in the retardation of AL elongation, a principal contributor to myopia development. The resultant relaxation of the ciliary muscles may prevent the AL elongation that typically initiates myopia, thus offering a preventative approach to this common refractive error (21).

RL and DIT have demonstrated the capacity to mitigate key stimuli contributing to the development of myopia, presenting as innovative approaches for its management. Given their distinct operational principles, they are capable of mutual reinforcement, with the potential for a synergistic effect when administered conjointly.

Clinical observations have corroborated that children subjected to efficacious interventions against environmental darkness and prolonged near work—such as engaging in outdoor activities for 6 h daily, adhering to a regimen of red light therapy, and utilizing distant-image screens over an extended period—have exhibited a reduction in myopia. Notably, a subgroup of children in the early stage of myopia showed improvement on SER. This underscores the imperative to investigate the therapeutic potential of myopia. By amassing a repertoire of efficacious myopia control strategies and countering each myopia-inducing stimulus effectively, we can discern the trajectory and likelihood of myopia regression, particularly in children with newly developed myopia.

The study’s findings underscore the efficacy of RL in slowing AL elongation. When RL is used in combination with DIT, compared with the sole use of RL, there is no significant difference in the changes in AL, yet there is a discernible shift in the SER towards hyperopia, suggesting a beneficial effect of DIT on myopia progression. The amalgamation of these two modalities is even more pronounced, with instances of minor diopter reduction and a case of −1.0D myopia reverting to emmetropy. This indicates that a comprehensive strategy of prevention and control holds substantial potential in the realm of myopia management.

The present study validates RL and DIT as effective means for myopia prevention and control, suggesting that their combined use may yield better results. This revelation introduces a novel paradigm for myopia management, especially pertinent in settings characterized by diminished outdoor engagement and heightened indoor near-vision demands, where an integrated approach to prevention and control is exceedingly valuable.

## Conclusion

The combined use of RL and DIT has shown good effectiveness in controlling myopia. Future studies should focus on examining the long-term effects and safety of this approach. These investigations will help develop better methods for managing myopia.

### Strength and limitations

The advantage of this study is the use of a randomized controlled trial design that helps eliminate many potential confounding factors. In addition, standardized refractive examination after cycloplegia improves the accuracy of efficacy evaluation. The limitations lie in two aspects. Firstly, the follow-up period of only 12 months makes it impossible to assess the long-term effects of interventions (such as myopic rebound or sustained control effects). Secondly, due to the obvious difference in appearance between DIT equipment and RL equipment, masking method cannot be implemented in this study. However, since the main outcomes are measured through objective examinations, the measurement bias may exist, but should be relatively small.

## Data Availability

The raw data supporting the conclusions of this article will be made available by the authors, without undue reservation.

## References

[1] Saw SM Wu HM Seet B Wong TY Yap E Chia KS. Academic achievement, close up work parameters, and myopia in Singapore military conscripts. Br J Ophthalmol. (2001) 85:855–60. doi: 10.1136/bjo.85.7.855, PMID: 11423462 PMC1724036

[2] Wen L Cao Y Cheng Q Li X Pan L Li L. Objectively measured near work, outdoor exposure and myopia in children. Br J Ophthalmol. (2020) 104:1542–7. doi: 10.1136/bjophthalmol-2019-315258, PMID: 32075819 PMC7587221

[3] Tian L Cao K Ma DL Zhao SQ Lu LX Li A. Investigation of the efficacy and safety of 650 nm low-level red light for myopia control in children: a randomized controlled trial. Ophthalmol Ther. (2022) 11:2259–70. doi: 10.1007/s40123-022-00585-w, PMID: 36208391 PMC9587157

[4] Xiong R Zhu Z Jiang Y Wang W Zhang J Chen Y. Longitudinal changes and predictive value of choroidal thickness for myopia control after repeated low-level red-light therapy. Ophthalmology. (2023) 130:286–96. doi: 10.1016/j.ophtha.2022.10.002, PMID: 36240954

[5] Zhou L Xing C Qiang W Hua C Tong L. Low-intensity, long-wavelength red light slows the progression of myopia in children: an Eastern China-based cohort. Ophthalmic Physiol Opt. (2022) 42:335–44. doi: 10.1111/opo.12939 34981548

[6] Zhen Y Zhang W Shen J Cheng DW Shen WR Wang NL. The clinical value of using a distant-image screen for reading and learning. Zhonghua Yan Ke Za Zhi. (2022) 58:1045–50. doi: 10.3760/cma.j.cn112142-20220106-00004 36480886

[7] Chen Z Zhou J Xue F Qu X Zhou X. Two-year add-on effect of using low concentration atropine in poor responders of orthokeratology in myopic children. Br J Ophthalmol. (2022) 106:1069–72. doi: 10.1136/bjophthalmol-2020-317980, PMID: 33707188

[8] Tomiyama ES Berntsen DA Richdale K. Peripheral refraction with toric orthokeratology and soft toric multifocal contact lenses in myopic astigmatic eyes. Invest Ophthalmol Vis Sci. (2022) 63:10. doi: 10.1167/iovs.63.8.10, PMID: 35819285 PMC9287617

[9] Yam JC Jiang Y Lee J Li S Zhang Y Sun W. The association of choroidal thickening by atropine with treatment effects for myopia: two-year clinical trial of the low-concentration atropine for myopia progression (LAMP) study. Am J Ophthalmol. (2022) 237:130–8. doi: 10.1016/j.ajo.2021.12.014, PMID: 34942105

[10] Ye L Xu H Shi Y Yin Y Yu T Peng Y. Efficacy and safety of consecutive use of 1 and 0.01% atropine for myopia control in Chinese children: the atropine for children and adolescent myopia progression study. Ophthalmol Ther. (2022) 11:2197–210. doi: 10.1007/s40123-022-00572-1, PMID: 36175821 PMC9521881

[11] Beasley IG Davies LN Logan NS. The effect of peripheral defocus on axial growth and modulation of refractive error in hyperopes. Ophthalmic Physiol Opt. (2022) 42:534–44. doi: 10.1111/opo.12951, PMID: 35187687 PMC9303555

[12] Zhang HY CSY L Tang WC Leung M To CH. Defocus incorporated multiple segments spectacle lenses changed the relative peripheral refraction: a 2-year randomized clinical trial. Invest Ophthalmol Vis Sci. (2020) 61:53. doi: 10.1167/iovs.61.5.53, PMID: 32460315 PMC7405698

[13] He M Xiang F Zeng Y Mai J Chen Q Zhang J. Effect of time spent outdoors at school on the development of myopia among children in China: a randomized clinical trial. JAMA. (2015) 314:1142–8. doi: 10.1001/jama.2015.10803, PMID: 26372583

[14] Huang J Wen D Wang Q McAlinden C Flitcroft I Chen H. Efficacy comparison of 16 interventions for myopia control in children: a network meta-analysis. Ophthalmology. (2016) 123:697–708. doi: 10.1016/j.ophtha.2015.11.010, PMID: 26826749

[15] Yam JC Li FF Zhang X Tang SM Yip BHK Kam KW. Two-year clinical trial of the low-concentration atropine for myopia progression (LAMP) study: phase 2 report. Ophthalmology. (2020) 127:910–9. doi: 10.1016/j.ophtha.2019.12.011, PMID: 32019700

[16] Cao K Tian L Ma DL Zhao SQ Li A Jin ZB. Daily low-level red light for spherical equivalent error and axial length in children with myopia: a randomized clinical trial. JAMA Ophthalmol. (2024) 142:560–7. doi: 10.1001/jamaophthalmol.2024.0801, PMID: 38662345 PMC11046409

[17] Rozema J Dankert S Iribarren R Lanca C Saw SM. Axial growth and lens power loss at myopia onset in Singaporean children. Invest Ophthalmol Vis Sci. (2019) 60:3091–9. doi: 10.1167/iovs.18-26247, PMID: 31323091

[18] Chiang ST Phillips JR Backhouse S. Effect of retinal image defocus on the thickness of the human choroid. Ophthalmic Physiol Opt. (2015) 35:405–13. doi: 10.1111/opo.12218 26010292

[19] Yi Z Jie G Wei Z Dewen C Wenrui S Ningli W. Prevention efficacy of desktop virtual reality display on digital eye strain. Ophthalmol China. (2022) 31:225–30. doi: 10.13281/j.cnki.issn.1004-4469.2022.03.011

[20] Delshad S Collins MJ Read SA Vincent SJ. The time course of the onset and recovery of axial length changes in response to imposed defocus. Sci Rep. (2020) 10:8322. doi: 10.1038/s41598-020-65151-5, PMID: 32433541 PMC7239843

[21] Meng ZY Yang L Zhou P. Ciliary muscles contraction leads to axial length extension—the possible initiating factor for myopia. PLoS One. (2024) 19:e0301844. doi: 10.1371/journal.pone.0301844, PMID: 38626193 PMC11020782

