# Supplementary material for: One-year changes in axial length and refraction in children using low-level red light and distant-image screen for myopia control: a randomized controlled trial
Source: Front Med (Lausanne). 2025 Mar 25;12:1542620. doi: 10.3389/fmed.2025.1542620 (PMC11977516; doi:10.3389/fmed.2025.1542620)
Supplement: Supplementary file 1 [file Image_1.pdf]

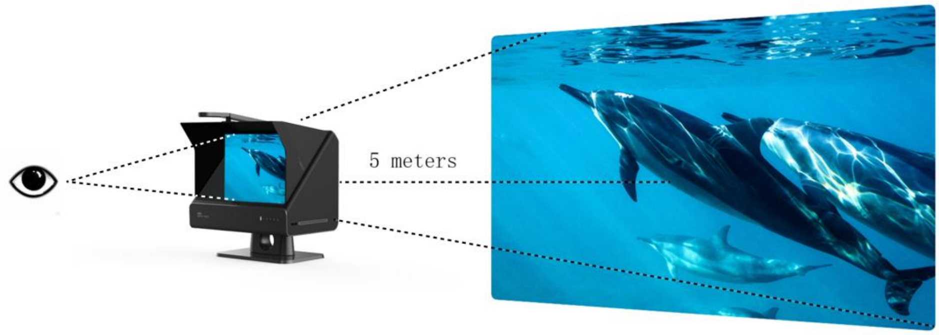

eFigure 1: Illustration of distant-image screen

The distant image screen employs virtual telephoto technology to project the image onto the screen as if it were located 5 meters away. Thus, even though the screen is placed in the same manner as that of ordinary electronic products, the perceived image appears to be at a distance of 5 meters.
